# Collagen Film with Bionic Layered Structure and High Light Transmittance for Personalized Corneal Repair Fabricated by Controlled Solvent Evaporation Technique

**DOI:** 10.3390/jfb13020052

**Published:** 2022-05-02

**Authors:** Peihong Ji, Chuanlei Zhang, Yanhui Kong, Huiyu Liu, Jia Guo, Longsheng Shi, Hui Yang, Zhongwei Gu, Yang Liu

**Affiliations:** 1 Research Institute for Biomaterials, Tech Institute for Advanced Materials, College of Materials Science and Engineering, Nanjing Tech University, Nanjing 211816, China; jpjia@163.com (P.J.); zwgu1006@hotmail.com (Z.G.); 2 Institute of Biomedical Engineering and Health Sciences, Changzhou University, Changzhou 213164, China; zhangcl@cczu.edu.cn (C.Z.); kongyh@cczu.edu.cn (Y.K.); liuhy@cczu.edu.cn (H.L.); guojia@cczu.edu.cn (J.G.); 3 Hangzhou Matrix Medical Technology Co., Ltd., Hangzhou 311100, China; shils@njtech.edu.cn; 4 Key Laboratory of Prevention and Treatment of Cardiovascular and Cerebrovascular Diseases, Ministry of Education, Gannan Medical University, Ganzhou 341000, China; yanghui_2521@gmu.edu.cn

**Keywords:** personalized corneal repair, bionic, collagen, solvent evaporation, multilayer structure

## Abstract

Corneal blindness is a common phenomenon, and corneal transplantation is an effective treatment for corneal defects. However, there is usually a mismatch between the corneal repair material and the degree of the patient’s corneal defect. Therefore, patients with different corneal defects need suitable corneal repair materials with a specific microstructure for personalized treatment. In this research, collagen films with bionic structures were fabricated through ethanol evaporation technique by regulating the volume ratios of collagen solution: ethanol = 10:0(Col)/9:1(CC91)/8:2(CC82)/CC73(CC73). Under various preparation conditions, the obtained collagen films contain layered structures of different density. SEM photos show that the CC73 film with a dense layer arrangement has a microstructure similar to that of the corneal epithelial layer, whereas the Col film has a loose layered structure similar to that of the corneal stroma layer. Four kinds of collagen films showed different optical properties and water absorption ability. A more ordered arrangement of internal layer structure leads to better mechanical properties of the collagen film. In view of this, we think that these collagen films with different microstructures and different interlayer spacing may have huge potential applications for personalized corneal repair.

## 1. Introduction

The native cornea tissue is an avascular, highly translucent, dome-shaped film that serves as a refractive and protective organ at the forefront of the eye [1]. Corneal defects, fungal infections and other causes often lead to corneal blindness, and keratoplasty remains the current gold standard for corneal visual rehabilitation [2,3]. The bioengineering of corneal repair materials that mimic native cornea tissue has attracted lots of interest owing to the scarcity of high-quality allogeneic corneal tissues for transplantation treatment of corneal blindness [4,5,6]. In recent years, reports on corneal repair substitutes have included various of biological materials, such as human amniotic membrane [7,8], natural collagen-based scaffolds [9,10,11,12], silk fibroin scaffold [13], chitosan and polysaccharide-based materials [14,15,16], etc. Collagen, as the main chemical component and the main load-bearing component of natural corneal tissue, has been extensively studied as a keratoprosthetics [17,18].

Histologically, the optically clear human cornea consists of five different parts: the corneal epithelium, anterior limiting lamina, corneal stroma, posterior limiting lamina, and corneal endothelium [19]. More than 90% of the cornea tissue is composed of a collagen-rich extracellular matrix within the cornea stroma, and the other 10% is composed of the corneal epithelium and endothelium layers and their corresponding supporting collagen layers. It is noteworthy that there are structural differences among the multilayered collagen matrix in the human cornea [20,21]. For example, the corneal epithelial layer and the endothelium layer have a dense layer structure, whereas the layer density of the corneal stromal is relatively loose [22]. At present, the defect site of patients with clinical keratopathy varies from person to person. However, most collagen-based material used for corneal repair has a uniform structure [23,24], which cannot meet the needs of some patients.

As we know, each part of the corneal tissue has a unique and subtle layered structure. Most physical characteristics of the cornea, including its mechanical properties, light transmittance, stability of shape, moisturizing ability, etc., are mainly attributed to the stratified structure of collagen in the corneal tissue [25,26]. Reproducing this structure on a nano and micro scale may be essential to engineer corneal tissue with functions similar to those of native cornea [27]. The aim of this study is to fabricate collagen films with different layered bionic structures by solvent evaporation technique to provide collagen-based implants for personalized corneal repair during severe donor cornea shortages.

## 2. Materials and Methods

### 2.1. Materials

Type I collagen (HM Biotech Ltd., Guangzhou, China) was obtained from bovine tendon. Ethanol was purchased from Guanghua Chemical (Shantou, China). 1-Ethyl-3-(3-dimethyl aminopropyl) carbodiimide (EDC, as cross-linker) and N-hydroxy succinimide (NHS, as catalyst) were supplied by GL Biochem (Shanghai, China). All cell-culture-related reagents in cell experiments were purchased from Sigma Chemical (St. Louis, MO, USA). Phosphate-buffered saline (PBS) was prepared from the standard tablet form (Calbiochem Corp, Novagen, Germany). Deionized water was obtained from a water purification system (Millipore S.A.S., France).

### 2.2. Preparation of Films

First of all, collagen was evenly dissolved in 0.01 mol/L HCl (7.5 mg/mL). Then, EDC and NHS were added to the obtained collagen solution and mixed at 4 °C thoroughly to form a solution with a mass ratio of EDC:NHS:Col = 0.5:0.5:6. The cross-linking reaction was performed by stirring the solution for 4 h. After that, a certain amount of ethanol (95%) was dripped into the collagen solution gently, with volume ratios of collagen solution: ethanol = 10:0(Col)/9:1(CC91)/8:2(CC82)/CC73(CC73). Afterwards, stirring was continued for 30 min, and the solution was transferred into a specific mold to form a corneal-shaped scaffold. The cross-linked collagen hydrogels were air-dried then and rinsed three times with deionized water.

### 2.3. Observation of Micro-Structure by Scanning Electron Microscopy

Cross sections of four collagen samples were observed by scanning electron microscopy. Before taking pictures, the SEM specimen was fixed, after which the specimens were coated with gold at 2–5 nm thickness in a sputter coater. Observations were performed with a ZEISS EVO 18 Oberkochen scanning electron microscope at 10 kV. Prior to comparison among images, three segments within each image were compared, with no significant differences between them.

### 2.4. Water Absorption of the Films

Swelling Properties of the films was measured by immersing them in PBS (pH = 7.4) at the normal physiological temperature of human cornea tissue. The wet weight of the films was measured immediately after gently blotting the film surface with filler paper to remove the absorbed water. The saturated water absorption was calculated according to the equation:

Water absorption = (W_t_ − W_0_)/W_t_ × 100%, (1)

Here, W_t_ represents the wet weight of the collagen films at target times, while W_0_ represents the initial dry weight of the films. The values are expressed as the mean ± standard error (n = 10).

As we know, the sizes of artificial cornea implants are different for each patient during keratoplasty, so the scaffolds used for cornea repair should be fabricate with various dimensions easily. Collagen films with known dimensions were swelled in PBS, and then thickness and surface area of the hydrated films were measured every hour. The thickness and surface area of the collagen films were measured by micrometer caliper and ruler, respectively. Variations in thickness and surface area were calculated according to the following equations:

Thickness increase = H_t/_H_0_, (2)

Surface area increase = S_t_/S_0_, (3)

Here, H_t_ and S_t_ are the thickness and surface area, respectively, of the wet samples at target times; and H_0_ and S_0_ are the initial thickness and surface area, respectively, of the dry films. The values are expressed as the mean ± standard error (n = 10).

### 2.5. Transmittance and Refractive Index

After equilibrium swelling was achieved, the transmittance of the four samples in the range of visual wavelength (350–800 nm) was analyzed by a ultraviolet–visible spectrophotometer (UV3802, Shanghai UNICO, China). A VEE GEE refractometer (Model C10) was used to quantify the refractive index of the collagen films. The films were placed on the surface of the primary prism after calibration using deionized water. After the adjustment of the crosshairs and the boundary line, the refractive index was recorded.

### 2.6. Mechanical Test

Mechanical tensile experiments were conducted according to the reported methods [9]. The stretch-strain characteristics of the wet collagen samples were tested using a uniaxial load instrument under a rate of 1 mm/min. After equilibrium swelling in PBS was achieved, the collagen samples (1.0 cm width × 2.0 mm length × 0.108 mm thickness) were clamped for axial tensile testing (n = 6). 

### 2.7. Diffusion Coefficient of the Films

Diffusion properties of the four collagen films relative to NaCl solution and tryptophan solution were measured using a specific device consists of two compartment chambers. The films were fixed between the permeate chamber (filled with either NaCl solution or tryptophan solution) and receptor chamber (filled with deionized water), and the concentrations of ions or tryptophan in the receptor chamber were subsequently checked. Ionic conductivity of the NaCl solution in the receptor chamber was determined by a DDS-11A conductivity meter (Jinmai, Shanghai, China). Colorimetric measurements of the tryptophan solution in the receptor chamber were performed at 540 nm using a tryptophan assay kit (GAG020, Sigma–Aldrich) with a UV3802 ultraviolet–visible spectrophotometer (Shanghai UNICO, China). The ions or tryptophan diffusion coefficient of the samples were acquired according to the method reported in [17].

### 2.8. Human Corneal Epithelial Cells and Corneal Stromal Cells Proliferation to the Film

In this study, the human corneal epithelial cells (HCECs) and human corneal stromal cells (HCSCs) were provided by the State Key Lab of Ophthalmology, Zhongshan Ophthalmic Center, China. Two kinds of cells were cultured separately in high-glucose Dulbecco’s Modified Eagle Medium (DMEM; Gibco BRL) containing 15% fetal bovine serum (Sijiqing, China), 2 mM L-glutamine, 5 μg mL^−1^ insulin, 5 μg mL^−1^ human transferrin, 100U mL^−1^ penicillin, 10 ng mL^−1^ human epidermal growth factor (Gibco BRL), and 100 μg mL^−1^ streptomycin (HyClone) at 5% carbon dioxide and 37 °C.

Before the cell experiments, all of the collagen films were washed more than three times in PBS under aseptic conditions, and then sterilized by ultraviolet radiation for 2 h and washed again in PBS. After sterilization, the collagen samples were transferred to tissue culture plates (Corning, UK). Next, the cell-seeded films were incubated in a humidified atmosphere (5% CO_2_ and 37 °C). The cell-culture medium was replaced every 48 hours. The response of HCECs to the Col, CC91, CC82, and CC73 films and the morphology of cells were observed. Before observation by inverted fluorescence microscopy (Olympus IX-70, Japan), the surface of the collagen films was washed gently with PBS. The rate of growth of the HCECs and HCSCs on the samples was determined quantitatively by methylthiazol tetrazolium (MTT) assay with a microplate reader at the optical density value of 490 nm.

### 2.9. Statistical Analysis

All data are shown as mean ± standard deviation. Data were analyzed using analysis of variance (ANOVA) to determine the significant differences among the groups. Statistical significance was defined as *p* < 0.05.

## 3. Results and Discussion

**Figure 1:**
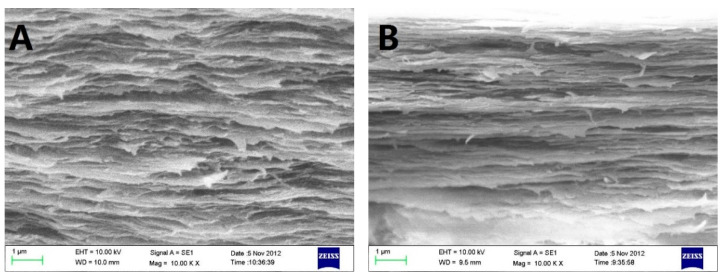

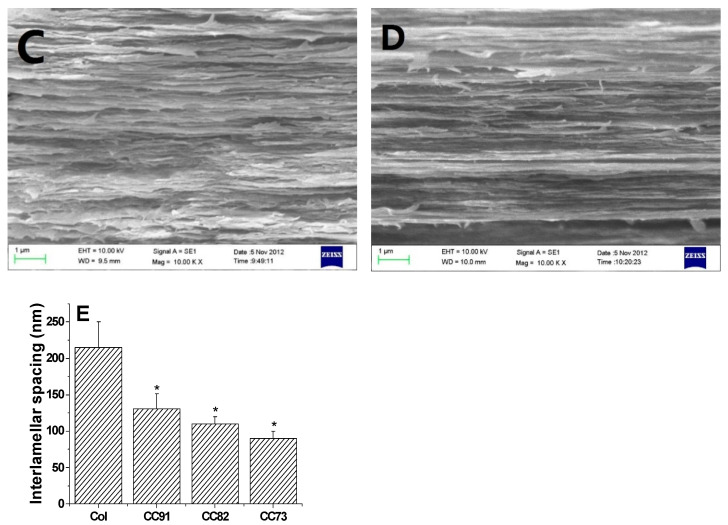
In vivo cross-section evaluation by scanning electron microscopy. SEM images of Col (**A**), CC91 (**B**), CC82 (**C**), and CC73 (**D**). Interlamellar spacing of the four collagen films (**E**) (n = 10). Asterisks represent significant differences between the experimental groups and the Col group. The CC73 film with dense layer arrangement has a microstructure similar to that of the corneal epithelial layer, whereas the Col film has a loose layered structure similar to that of the corneal stromal layer. * significant differences between the experimental groups and the Col group.

Scanning electron micrograph (SEM) photos of layered structure of the Col, CC91, CC82m and CC73 film are displayed in Figure 1. All four collagen samples have a good, layered structure. However, the layered structure of the Col film (Figure 1A) is significantly looser than that of the other three samples (Figure 1B–D). With increasing alcohol content, the interlayer structure of the samples becomes compact. This may because ethanol reduced the interfacial tension between the collagen molecule and the tiny water bubbles. Compared with the evaporation rate of water in Col solution, water in CC91, CC82, and CC73 solution was evaporated faster with ethanol evaporation, which makes the CC91, CC82, and CC73 films more compact. Figure 1E shows that the approximate interlamellar spacings of the four collagen films are different (215 ± 35 nm for Col, 131 ± 20 nm for CC91, 110 ± 10 nm for CC82, and 90 ± 11 nm for CC73). Similar to the acellular porcine corneal stroma (APCS) used for rabbit corneal repair [10,11,28], APCS with different structures can be used for corneal tissue repair in different animals. Therefore, the obtained dense collagen films may have potential applications for corneal epithelial or endothelial repair, whereas the loose Col sample can be used for stromal repair.

**Figure 2:**
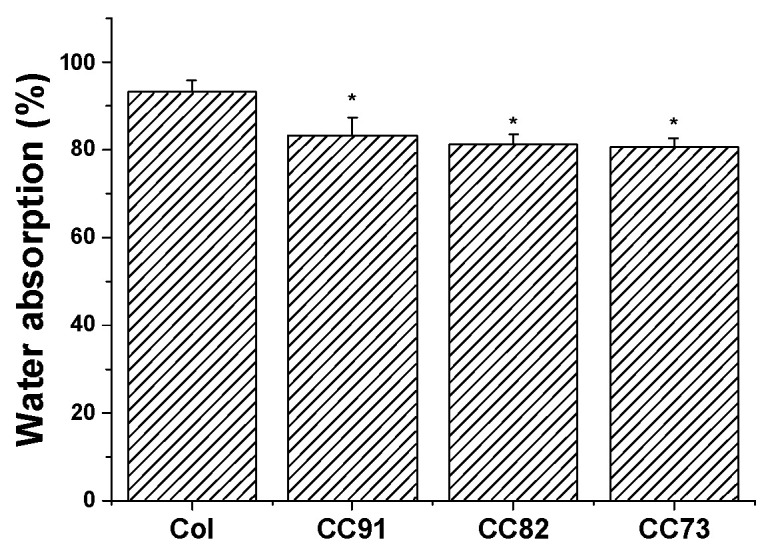
Water absorption percentages of the Col, CC91, CC82, and CC73 membrane (n = 10). The larger internal space inside the Col film results in greater water absorption capacity than CC91, CC82, and CC73 films. * significant differences between the experimental groups and the Col group.

Moisturizing performance of the cornea is an important indicator of normal tissue, so the saturated water-absorption and swelling properties of corneal repair materials are very important. Figure 2 shows the water absorption of the four collagen samples. After the films were immersed in PBS for 1 h, saturated water absorption of the samples tended to be constant. Saturated water absorption of Col, CC91, CC82, and CC73 film was (93.2 ± 2.7)%, (83.4 ± 3.9)%, (81.3 ± 2.3)%, and (80.7 ± 2.1)%, respectively. The absorption capacity of the Col film was higher than that of CC91, CC82, and CC73, possibly because the interlayer structure of the Col film is looser than that of the other three samples. The larger internal space inside the Col film results in greater moisture storage capacity.

**Figure 3:**
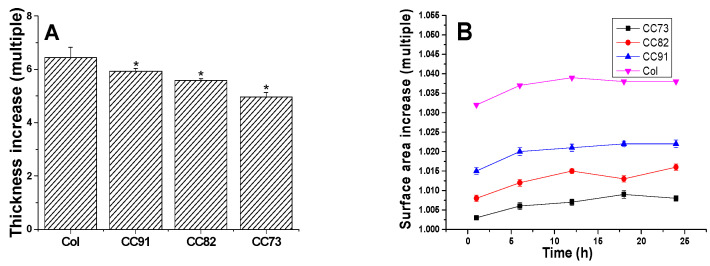
Variation of the Col, CC91, CC82, and CC73 samples’ thickness (**A**) and surface area (**B**) in PBS. Values are expressed as the mean ± standard deviation (n = 10). These collagen films could easily be fabricated with various internal structures and different dimensions due to their stable morphological changes after absorbing water. * significant differences between the experimental groups and the Col group.

Figure 3A shows the thickness increase of the Col, CC91, CC82, and CC73 films. After the samples were immersed in PBS for 1 h, water absorption of the films tended to be constant. The thickness of the four films increased more than five times. The thickness change of CC91 and CC82 was lower than that of Col but slightly higher than that of CC73. Figure 3B shows the variation of the four samples’ surface area versus time. The sizes of the four collagen films changed slightly after water absorption. The surface area changes of CC91, CC82, and CC73 were lower than that of Col. This may also be due to the different size of the space inside the films, resulting in different structural changes after water absorption. According to the above results, we concluded that these collagen films could easily be fabricated with various internal structures and different dimensions.

**Figure 4:**
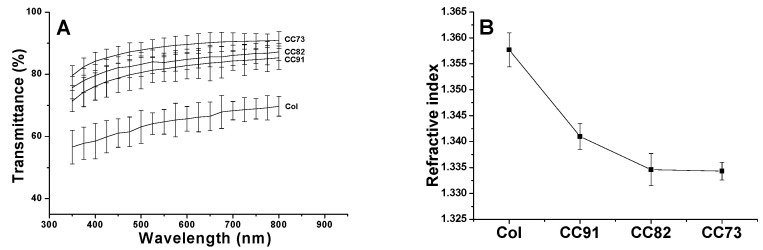
Light transmittances of the Col, CC91, CC82, and CC73 films in the range of 350–800 nm (**A**). Refractive index of collagen films (**B**) (n = 10). With the decrease in the interlayer spacing of the collagen films, the light transmittance of the material increases gradually.

Optical properties directly influence the effect of corneal repair materials. The light transmittance curve of the four samples is shown in Figure 4A. With the increase in alcohol content in the collagen sample, the luminousness of the collagen films increased. In addition, with the increase in wavelength, the light transmittance of the CC73 film increased to its maximum (higher than 90%), which tended to be constant in the range of visible light. Figure 4B indicates the refractive index of Col, CC91, CC82, and CC73 film is 1.3577 ± 0.0033, 1.341 ± 0.0025, 1.3346 ± 0.0031, and 1.3343 ± 0.0017, respectively. With the increase in ethanol content, the refractive index of collagen films decreased. These water-absorbing collagen films contain two phases: a water phase and a collagen phase. When the refractive index of the film is closer to water, the scattering of light in the wet sample is weakened [14], and therefore, the light that passes through the material is more conductive. Based on the results of light transmittance and refractive index measurements, the CC73 film has the best optical performance.

**Figure 5:**
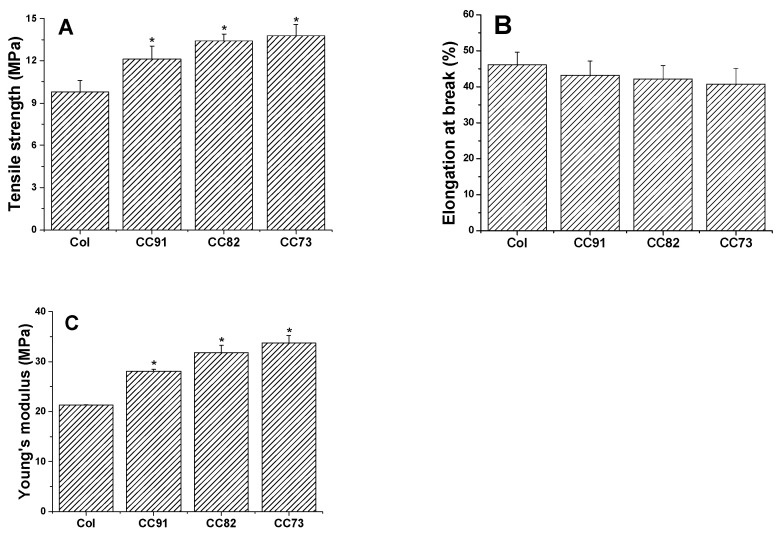
Mechanical properties of the Col, CC91, CC82, and CC73 films (n = 6). Asterisks represent the significant differences between the experimental groups and the Col group. The mechanical properties of the films as follows: tensile strength (**A**), elongation at break (**B**), and Young’s modulus (**C**). With the decrease in the interlayer spacing of the collagen films, the tensile strength and Young’s modulus of the materials increase gradually. * significant differences between the experimental groups and the Col group.

Mechanical performance is one of the key factors of collagen-based corneal repair materials. The good mechanical properties of the artificial cornea material can meet the requirements of the ocular surface to maintain normal intraocular pressure. As shown in Figure 5, a comparison between the four collagen samples shows that they exhibit different mechanical behavior. The ultimate tensile strength of the four films is 9.81 ± 0.78 MPa for Col, 12.13 ± 0.91 MPa for CC91, 13.41 ± 0.49 MPa for CC82, and 13.76 ± 0.82 MPa for the CC73 film (Figure 5A). The ultimate tensile strength of CC91, CC82, and CC73 films is higher than that of Col film. However, there was no significant difference in elongation at break between the four samples (Figure 5B). Furthermore, on the basis of the above work, the Young’s modulus is 21.27 ± 0.07 MPa for Col, 28.07 ± 0.43 MPa for CC91, 31.79 ± 1.50 MPa for CC82, and 33.77 ± 1.46 MPa for CC73 (Figure 5C). The results indicate that the dense films have better mechanical properties than the loose samples. When the collagen film is stretched, the material with a dense multilayer structure shows better tolerance in the direction of the force of action.

**Figure 6:**
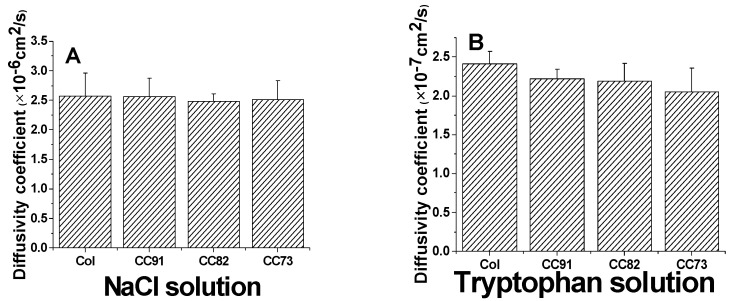
Ion solution diffusion (**A**) and tryptophan permeability (**B**) of the Col, CC91, CC82, and CC73 films (n = 6). All four collagen samples are permeable to both NaCl solution and tryptophan solution.

Because the cornea is an avascular tissue, the nutrients for cornea cells to maintain their normal function mainly depend on the diffusion of aqueous humor, so a suitable and stable diffusion coefficient of the cornea repair materials is also required. The results of a diffusion test indicate that all of the collagen samples are permeable to both NaCl solution and tryptophan solution (Figure 6). According to the Fick’s law, the relationship of the ion solution (tryptophan) concentration of the receptor chamber with the diffusion time can be described according to the following formulas:

(4) $$-\ln(1-\frac{2C}{C_{0}})=\frac{2PS}{Vd}t,$$

(5) $$P=\frac{-\ln(1-\frac{2C}{C_{0}})Vd}{2St},$$

where P denotes the diffusion coefficient; t is the diffusion time; d is the thickness of the wet film; V and S are the volume of the solution in chamber and the round through-hole area between the chambers, respectively; C_0_ is the initial ion concentration of the permeate chamber; and C is the ion concentration of the receptor chamber at the target time.

The area of through-hole between the chambers was about 3.14 cm^2^ (S). The volumes of the experiment solutions in the two-compartment chambers were both 250 mL (V), and the thickness of the wet samples was 110 ± 5µm (d). The diffusion coefficient of the sample to NaCl is about (2.57 ± 0.39) × 10^−6^ cm^2^/s for Col, (2.56 ± 0.31) × 10^−6^ cm^2^/s for CC91, (2.48 ± 0.13) ×10^−6^ cm^2^/s for CC82, and (2.51 ± 0.32) × 10^−6^ cm^2^/s for CC73 film (Figure 6A). The diffusivity of the four samples is similar as the human corneal’s permeability coefficient, which is about 2.5 × 10^−6^ cm^2^/s [29]. The diffusion coefficient of the films to tryptophan is about (2.41 ± 0.16) × 10^−7^ cm^2^/s for Col, (2.22 ± 0.12) × 10^−7^ cm^2^/s for CC91, (2.19 ± 0.23) × 10^−7^ cm^2^/s for CC82, and (2.05 ± 0.31) × 10^−7^ cm^2^/s for CC73 (Figure 6B). As we know, the nutrients’ diffusion speed is related to the space structure of the scaffolds. With the increase in internal space size of the samples, the diffusion coefficient increased evidently.

**Figure 7:**
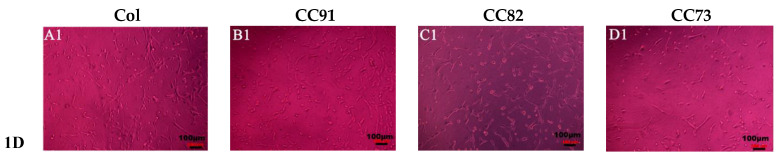

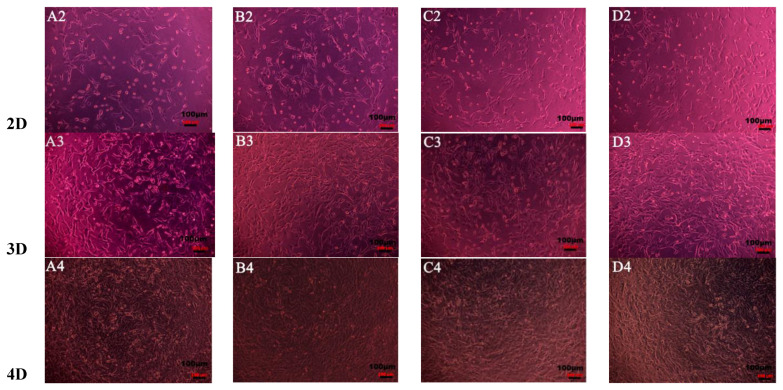
Morphology and proliferation of HCECs on the Col (**A1**–**A4**), CC91 (**B1**–**B4**), CC82 (**C1**–**C4**), and CC73 (**D1**–**D4**) at different time points (scale bar = 100 μm). The cells were incubated for 24 h (**A1**–**D1**), 48 h (**A2**–**D2**), 72 h (**A3**–**D3**), and 96 h (**A4**–**D4**). After incubation, the HCECs attached and grew well on the four collagen films. Within the 48 h (**A2**–**D2**), the morphology of the seeded cells gradually changed from a round shape to a spindle shape.

Figure 7 shows the shape and proliferation of HCECs on the Col, CC91, CC82, and CC73 films at different time points. We can found that, after the cells were incubated for 24 h (Figure 7A1–D1), 48 h (Figure 7A2–D2), 72 h (Figure 7A3–D3), and 96 h (Figure 7A4–D4), the human cornea epithelial cells attached and grew well on the four collagen films. Within the 48 h, the seeded cells adhered to the surface of the film quite well. The shape of HCECs was gradually changed from a round shape to a spindle shape, which is quite similar to the HCECs grows on the normal cornea tissues.

**Figure 8:**
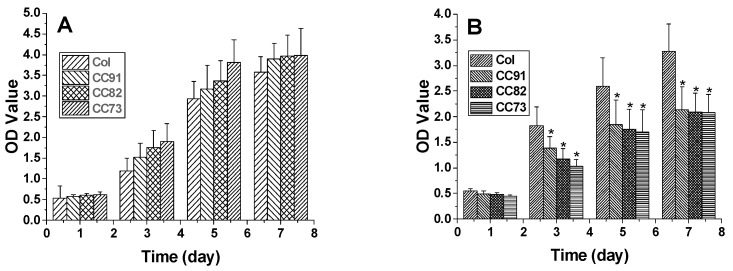
The proliferation of human corneal epithelial cells (**A**) and human corneal stromal cells (**B**) on the Col, CC91, CC82, and CC73 membranes (n = 10) (MTT measured in units of optical density at 490 nm). * significant differences between the experimental groups and the Col group.

In this research, we chose two kinds of corneal cells to evaluate the biocompatibility of the four samples. The results indicate that all the four materials have good biocompatibility. Figure 8A shows the proliferation rate of human corneal epithelial cells (HCECs) on the Col, CC91, CC82, and CC73 films. HCECs proliferated rapidly after they were seeded on these films. After the HCECs were seeded, they had a similar growth rate on the first day. However, the HCEC growth rate on the Col film was slightly lower than that of the HCEC grown on CC91, CC82, and CC73 in the subsequent few days. Figure 8B shows the proliferation of human corneal stromal cells (HCSCs) on the Col, CC91, CC82, and CC73 samples. As the layer space increased, the rate of cell proliferation increased. We believe that the internal space structure of the scaffolds is closely related to oxygen and nutrient diffusion, which facilitated the HCSCs’ adhesion and proliferation.

## 4. Discussion

Currently, although treatment of corneal blindness with keratoplasty has a high success rate, the major problem is the shortage of high-quality donor tissues in most countries. Another problem is that the donated corneal tissue often does not exactly match the topography of the patient’s ocular surface [3,4]. For synthetic corneal substitutes, the problem is that the microstructure and size of the corneal repair materials used for different corneal blind patients with different defect sites are usually the same [2,7,8,15]. However, patients with corneal epithelial defects and corneal stromal defects need artificial corneas with different structures for better repair. Transparent films prepared by collagen I have demonstrated significant potential for cornea repair and may therefore provide a suitable substitute for donor cornea [9,17,18]. In this study, collagen films with different layered bionic structures were fabricated by regulating the volatilization process of ethanol. Compared with the evaporation rate of water in Col solution, water in CC91, CC82, and CC73 solution is evaporated faster with ethanol evaporation, which makes the multilayer structure of CC91, CC82, and CC73 films more compact. Similar to the acellular porcine corneal stroma (APCS) used for corneal repair in rabbit models [10,11,28], the APCS with dense structure can be used for corneal epithelial repair [10], and the APCS with loose structure can be used to repair the corneal stroma [11]. Therefore, we think the obtain dense collagen films may have potential applications in corneal epithelial or endothelial repair, whereas the loose Col sample can be used for stromal repair.

In developing artificial cornea materials, we are concerned with function and biocompatibility [17]. In order to improve the function, the implant must possess appropriate properties, such as mechanical strength, stability of shape, moisturizing ability, and light transmittance, just to name a few [20,21,28,30]. As we know, each part of the corneal tissue has a unique, subtle, layered structure. Most physical characteristics of the cornea are attributed to the stratified structure of collagen in the corneal tissue. The absorption capacity of Col is higher than that of CC91, CC82, and CC73; this may be because the interlayer structure of the Col is looser than that of the other three samples. Larger internal space inside the Col film results in greater moisture storage capacity. Based on the stable correspondence between the morphologies of these collagen films before and after saturated water absorption, we believe that they can be easily fabricated with various dimensions [9]. It is noteworthy that the light transmittance of the collagen films increases with the gradually decrease in the interlayer spacing. To examine the mechanical properties of Col, CC91, CC82, and CC73 films, we measured the ultimate tensile strength, elongation at break, and Young’s modulus. The ultimate tensile strength of CC91, CC82, and CC73 films is slightly higher than that of the native corneal tissue (11 ± 0.5 MPa) [9,18]. The tensile strength and Young’s modulus of the film increases as the film’s structural density increases. With the decrease in the interlayer spacing of the films, the free volume of the functional groups of the cross-linked collagen molecules is decreased, the steric hindrance is increased, and molecular deformation energy is increased, leading to the high tensile strength and elastic modulus of collagen film at the macro level [9,17]. MTT tests demonstrate that the four collagen films have no cytotoxic reactivity against human corneal epithelial cells and corneal stromal cells. The morphology of HCECs on these films is quite similar to that of HCECs grown on normal cornea tissue [5,9,14]. The epithelialization process of HCECs on the surface of collagen films can basically be completed within 96 h. The result of the cell experiments shows that all four films have good biocompatibility. The repair effect of these collagen films in vivo will be evaluated in future studies.

## 5. Conclusions

In this study, we prepared collagen films with layered bionic structures through a simple solvent evaporation technique. These collagen films fabricated under different conditions have various bionic microstructures, resulting in different optical properties, water absorption ability, and mechanical properties. The obtained dense multilayered collagen films may have potential applications for corneal epithelial or endothelial repair, whereas the loose multilayered collagen films can be used for corneal stromal repair. The results indicate that these collagen-based films may have potential applications for personalized corneal repair. In addition to corneal repair, we also believe that these collagen films have application potential for other soft tissue repair.

## Data Availability

Data are included in the text; raw data are available from the corresponding authors.
